# The role of cytochrome *c* oxidase subunit Va in non-small cell lung carcinoma cells: association with migration, invasion and prediction of distant metastasis

**DOI:** 10.1186/1471-2407-12-273

**Published:** 2012-06-29

**Authors:** Wen-Liang Chen, Kuang-Tai Kuo, Teh-Ying Chou, Chien-Lung Chen, Chih-Hao Wang, Yau-Huei Wei, Liang-Shun Wang

**Affiliations:** 1 Department of Biological Science and Technology, National Chiao Tung University, Hsinchu, Taiwan; 2 Division of Thoracic Surgery, Department of Surgery, Shuang Ho Hospital, Taipei Medical University, Taipei, Taiwan; 3 Translational Research Laboratory, Shuang Ho Hospital, Taipei Medical University, Taipei, Taiwan; 4 Department of Pathology, Taipei Veterans General Hospital, Taipei, Taiwan; 5 Institute of Clinical Medicine, National Yang-Ming University, Taipei, Taiwan; 6 Institute of Biochemistry and Molecular Biology, National Yang-Ming University, Taipei, Taiwan; 7 Graduate Institute of Clinical Medicine, Taipei Medical University, Taipei, Taiwan

**Keywords:** Non-small cell lung cancer, Cytochrome *c* oxidase subunit Va, Migration, Invasion

## Abstract

**Background:**

Lung cancer is one of the most lethal malignancies worldwide, but useful biomarkers of lung cancer are still insufficient. The aim of this study is to identify some membrane-bound protein(s) associated with migration and invasion in human non-small cell lung cancer (NSCLC) cells.

**Methods:**

We classified four NSCLC cell lines into high and low migration/invasion groups by Transwell and Matrigel assays. Using two-dimensional gel electrophoresis and matrix-assisted laser desorption ionization-time of flight mass spectrometry (MALDI-TOF MS), we identified 10 membrane-associated proteins being significantly overexpressed in the high migration/invasion group. The expression of the target protein in the four NSCLC cell lines was then confirmed by reverse transcription polymerase chain reaction (RT-PCR), western blot and immunostaining. RNA interference technique was applied to observe the influence of the target protein on migration and invasion. Gelatin zymography was also performed to evaluate the activities of matrix metalloproteinase (MMP)-2 and MMP-9. Expression condition of the target protein on surgical specimens was further examined by immunohistochemical staining and the clinicopathologic data were analyzed.

**Results:**

We identified a mitochondria-bound protein cytochrome *c* oxidase subunit Va (COX Va) because of its abundant presence found exclusively in tumorous areas. We also demonstrated that migration and invasion of NSCLC cells decreased substantially after knocking down COX Va by siRNA. Meanwhile, we found a positive correlation between COX Va expression, Bcl-2 expression and activities of MMP-2 and MMP-9 in NSCLC cells. Immunohistochemical staining of surgically resected lung adenocarcinomas in 250 consecutive patients revealed that strong COX Va expression was found in 54.8% (137/250) of patients and correlated positively with the status of lymph node metastasis (*P* = 0.032). Furthermore, strong COX Va expression was associated with the presence of distant metastasis (*P* = 0.033).

**Conclusions:**

Our current study showed that COX Va may play a role in migration and invasion of NSCLC cells and can be used as a biomarker to predict aggressiveness of NSCLC.

## Background

Lung cancer, predominantly non-small cell lung cancer (NSCLC), is currently the leading cause of male cancer-related death worldwide [1]. Despite application of multimodal treatments, the overall survival of NSCLC patients remains poor [2-4]. About 40-50% patients of NSCLC present with stage IV disease [5], and given that complete surgical resection may provide a chance of cure in patients with early-stage tumors, the reported recurrence rate in the patients with completely resected stage I NSCLC was nearly 30% [6-8]. Moreover, among these patients with tumor recurrence, more than 70% of them have distant metastasis [6,8]. All these facts indicate that metastasis is the most frequently encountered problem in treating NSCLC. Metastasis is a complicated process with tumor invasion being the first step, followed by arrest in the blood stream, and finally metastatic colonization [9]. However, molecular factors that are related to invasion are still insufficient, and identification of such factors with elucidation of their molecular mechanism will provide insight into cancer biology and potentially provide new therapeutic targets for NSCLC patients.

The rapid development of proteomic technologies during the past 10 more years has brought about a massive increase in the discovery of novel cancer biomarkers. The biomarkers may have broad applications, such as for detecting the presence of a disease, monitoring the status of the disease, or evaluating the treatment response. The commonly used techniques to discover these biomarkers, also referred to as quantitative proteomics, are performed by protein separation using either two-dimensional gel electrophoresis (2DE)- or liquid chromatography (LC)-based methods coupled with protein identification using mass spectrometry (MS) [10]. Regarding lung cancer, Chen et al. used tissue samples and quantitative proteomics to identify some proteins involving in controlling gluconeogenesis and glycolysis that were associated with the survival of patients with early-stage lung adenocarcinoma [11]. Tian et al. applied NSCLC cell lines plus 2DE, matrix-assisted laser desorption ionization-time of flight mass spectrometry (MALDI-TOF MS) and tandem mass spectrometry to recognize S100A11 as an important regulatory molecule in promoting invasion and metastasis of NSCLC [12].

In the current study, we studied the non-secreted membrane-bound proteins that are associated with migration/invasion in NSCLC cells. We separated four NSCLC cell lines into high and low migration/invasion group, and then comparatively analyzed the membrane protein profiles between these two groups by conventional 2DE followed by MALDI-TOF MS. A subunit of cytochrome *c* oxidase (COX) called COX Va was chosen to be the target of further study because of its presence exclusively in tumorous areas but not in non-tumorous areas in surgical specimens of NSCLC. The correlations between COX Va expression and activities of matrix metalloproteinase-2 (MMP-2), matrix metalloproteinase-9 (MMP-9), and Bcl-2 expression were studied. The clinicopathologic significances of COX Va overexpression were also evaluated in surgically resected lung adenocarcinomas in 250 consecutive patients by immunohistochemical staining.

## Methods

### NSCLC cell lines, cell culture and surgically dissected tumor samples

The cell lines CL1-1 and CL1-5 were sublines with different invasiveness derived from the same parental line [13]. To increase the discrimination power of the study, we added two more NSCLC cell lines that exhibited different abilities of migration/invasion. Two human adenocarcinoma cell lines were purchased from American Type Culture Collection (ATCC) (Manassas, VA): H2126 (ATCC No. CCL-256) and H2009 (ATCC No. CRL-5911). The CL1-1 and CL1-5 were kind gifts from Professor Pan-Chyr Yang at National Taiwan University. Cells were cultured in the RPMI 1640 medium (GIBCO, Gaithersburg, MD) containing 10% fetal bovine serum (FBS) (Jacques Boy, Reims, France), 100 U/mL penicillin (GIBCO) and 100 U/mL streptomycin (GIBCO) at 37°C in a 5% CO_2_ atmosphere at 99% humidity. A total of 250 surgically resected tumor samples, confirmed to be adenocarcinoma by histopathologic examinations, were obtained from consecutive patients with NSCLC between 2000 and 2005. Written informed consents were obtained from the patients with the study protocol approved by the ethical committee of the hospital.

### Migration assay

Cell migration assays were performed using Boyden chamber assay. The procedures were performed according to the manufacturer’s recommended protocol and had been previously described [13]. Cells were harvested and suspended in RPMI 1640 medium containing 10% FBS at a concentration of 5 × 10^4^ cells/mL. A Transwell apparatus with 8 μm pore size membrane (Millipore, Billerica, MA) was used to analyze the migration activity. The suspended cells in 100 μL of serum-free RPMI 1640 medium were seeded onto the upper chamber of the apparatus and 250 μL of RPMI containing 10% FBS were added to the insert well followed by an incubation at 37°C for 6 hours. The inner side of the chamber was then wiped with a wet swab to remove the cells, while the outer side of the chamber was gently rinsed with PBS and subjected to Giemsa staining (Sigma-Aldrich, St. Louis, MO) for 10 minutes. The membrane was then rinsed with phosphate-buffered saline (PBS). After drying, the membrane was photographed followed by counting the cell number. Each cell line was evaluated in triplicate.

### Invasion assay

Invasion assay was performed by a modification of the method previously described [14]. One hundred μL Matrigel (Becton Dickinson, Franklin Lakes, NJ) diluted to 1 mg/mL in serum-free RPMI was added to each upper chamber of the Transwell apparatus. After solidification of Matrigel at 37°C, approximately 5 × 10^4^ cells in serum-free RPMI were seeded onto the Matrigel over the upper chamber, followed by an addition of 250 μL of RPMI containing 10% FBS at the bottom insert-well. After incubation of the cells at 37°C for 18 hours, the inner side of the chamber was wiped with a wet swab to remove the cells, while the outer side of the chamber was gently rinsed with PBS and stained with the Giemsa stain solution for 10 minutes. Finally, the membrane was rinsed and allowed to air-dry. The membrane was photographed followed by counting the cell number and each cell line was evaluated in triplicate.

### Preparation of protein extract from membranes

A total of 2 × 10^6^ each NSCLC cells were suspended and washed by PBS for three times, followed by centrifugation at 1,500 g for 5 minutes. Cell membranes were isolated and extracted by a Compartment Protein Extraction Kit (BioChain Institute, Hayward, CA) following the procedures described by the manufacturer.

### 2D Gel electrophoresis

Immobilized 13-cm linear pH gradient strips, pH 3-11, were rehydrated overnight in a rehydration buffer containing 7 M urea, 2 M thiourea, 4% CHAPS, 0.5% gradient buffer and 50 mM dithiothreitol (DTT) according to the manufacturer's instructions (Amersham Biosciences, Buckingham, UK). Isoelectrofocusing was performed at 20°C using an IPGphor apparatus (Amersham) with 50 mA for a total of 40 kV/h. Strips were equilibrated in 50 mM Tris–HCl, 6 M urea, 30% (v/v) glycerol, 1% (w/v) sodium dodecyl sulfate (SDS) and 65 mM DTT, pH 8.8 for 20 minutes in the same buffer containing 240 mM iodoacetamide. Equilibrated strips were transferred onto 15% uniform polyacrylamide gels poured between the two glass plates (18 × 20 cm). Strips were overlaid with 0.5% (w/v) low melting point agarose prepared in a running buffer containing bromphenol blue. Gels were run at 10°C using the Ettan Dalt 6 apparatus (Amersham) at 2.5 W/gel for 30 minutes with 30 mA in total until the dye front run off the bottom of the gels. All the images were collected on a Typhoon 9400 Variable Mode Imager (Amersham). Statistics and determination of protein expression levels were carried out by a Decyder software (Amersham).

### MALDI-TOF MS

Protein spots were excised manually from Coomassie blue-stained gels and washed twice in 25 nM NH_4_HCO_3_ for 10 minutes until the gel pieces become transparent. Gel pieces were dehydrated with 100% acetonitrile for 5 minutes and allowed to dry, followed by incubation with 15 μL of a 12.5 ng/μL trypsin solution at 37°C overnight. Trypsinized samples were then concentrated using a C18 Zip-Tips Kit (Millipore) and eluted with 50% acetonitrile/0.1% trifluroacetic acid according to the manufacturer’s instructions. Identification of peptides was performed using a Microflex MALDI-TOF LRF20 mass spectrometer (Bruker Daltonics, Billerica, MA). The Zip-Tips-purified peptide digests were spotted with a cyano-4-hydroxycinnamic acid matrix and run in reflection positive-ion mode at an accelerating voltage of 25 kV. Subsequently, protein identification was done using MASCOT search engine from SWISS-PROT database (http://www.matrixscience.com).

### Reverse transcription polymerase chain reaction (RT-PCR)

Level of mRNA expressions corresponding to COX Va and other genes in NSCLC cells were determined by RT-PCR. Total RNA was extracted from cells using a SNAP RNA column (Invitrogen Corporation, San Diego, CA). Following spectrophotometric determination of RNA yield, cDNA is synthesized with oligo(dT) primer using avian myeloblastosis virus (AMV) reverse transcriptase. An aliquot of cDNA was subjected to 35 cycles of PCR using a standard procedure denaturing at 94°C for 30 seconds, hybridizing at 57°C for 30 seconds and elongating at 72°C for 1 minute. Amplified products were resolved in a 1 % agarose gel and visualized by ethidium bromide staining. DNA sequences of the amplified fragment were determined by an automatic sequenator (ABI, Foster City, CA) with the nucleotide sequences matched with the database from GenBank (http://www.ncbi.nlm.nih.gov/blast). The primer sequences used were as follows: COX Va (forward) 5’- TCGCCGTCATGCTGGG -3’ and (reverse) 5’- CAATAAATCCTTGGGGAAGCC -3’, MMP-2 (forward) 5’- AGAAGGCTGTGTTCTTTGCAG -3’ and (reverse) 5’- AGGCTGGTCAGTGGCTTG -3’, MMP-9 (forward) 5’- ATCCGGCACCTCTATGGTC -3’ and (reverse) 5’- GCTCTGAGGGGTGGACAGT -3’, Bcl-2 (forward) 5’- ATGTGTGTGGAGAGCGTCAACC -3’ and (reverse) 5’- TGAGCAGAGTCTTCAGAGACAGCC -3’. The primer sequences for the house-keeping gene β-actin were (forward) 5’- CCAGATCATGTTTGAGACCT -3’ and (reverse) 5’- CAACTAAGTCATAGTCCGCC -3’.

### Western blot

SDS-PAGE containing 15% polyacrylamide (unless specified otherwise) was used to analyze the COX Va content using a modified procedure described previously [15]. Electrophoresis was conducted in a vertical slab gel unit (Mini PIII, Bio-Rad, Hercules, CA) equipped with a PAC 300 power supply (Bio-Rad). All samples (20 μg) for SDS-PAGE were equilibrated in 10 mM Tris-HCl and 5% SDS (pH 7.6) before loading onto the gel. Following electrophoresis, the gel was soaked briefly and instantly in a transfer buffer containing 25 mM Tris, 192 mM glycine, 20% methanol, and 0.0375% SDS (pH 8.3) for 30 seconds. The gel was then immediately electrotransferred to a nitrocellulose membrane (Hybond-ECL extra; Amersham) at 90 mA for 60 minutes in a semi-dry Transfercell (Bio-Rad). The membrane was immersed in 1% skin milk for 1 h with gentle shaking. Following three washes with PBS for 5 minutes each, the membrane was subjected to reaction with a mouse monoclonal antibody against COX Va (MitoScience, Eugene, OR) at a 1:1,000 dilution and developed with chemiluminescence according to the method described previously [15].

### COX Va gene knockdown

The small interfering RNA (siRNA) targeting human COX Va was designed using a BLOCK-iT RNAi Designer algorithm (http://rnaidesigner.invitrogen.com/rnaiexpress/). To avoid unintentional silencing of non-targeted host cell genes, sequence homology was checked using a basic local alignment search tool (BLAST) search (http://blast.ncbi.nlm.nih.gov/Blast.cgi). A scrambled-sequence oligonucleotide (CAAAGCAGGACCTCATAAGGAAATCTACC) was used as a negative siRNA control. Pairs of complementary oligonucleotides sequences were synthesized, annealed and cloned into the pRS shRNA vector (OriGene, Rockville, MD). NSCLC cells were transfected with one of the four shRNAs using TurboFect™ (Fermentas, Glen Burnie, MD) as described below. After 48 hours, COX Va expression was assessed using RT-PCR. The results showed that two COX Va siRNA (ACTTGTTACCTATGATATGGTTCCAGAGC and CCTATGTCATCCAGGAACTTAGACCAACT) possessed the highest activity to suppress the expression of COX Va, and were used for subsequent experiments.

### Transfection of siRNA

TurboFect™ was used to deliver plasmid DNA containing siRNA against human COX Va (shCOX Va) into NSCLC cells in a 24-well culture plate. In each well, approximately 5x10^4^ cells in 1 mL was seeded and grown for 24 hours prior to transfection. While gently shaking, 1 μg of plasmid in 100 μL of RPMI medium was slowly added into 2 μL of TurboFect™ and incubated at room temperature for 15-20 minutes. The reaction mixture was then transfected into cells, which were grown in the culture medium containing 2 μg/mL puromycin for another 3 days, while plasmid without shRNA was used as a negative control.

### Gelatin zymography

To determine the enzyme activities of MMP-2 and MMP-9, media derived from the NSCLC cells cultured for 48 hours were used for the gelatin zymography [16,17]. An aliquot of 10 μL culture medium was loaded onto a SDS-PAGE gel under non-reducing conditions using an 8% polyacrylamide gel containing 0.1 mg/mL gelatin. After electrophoresis, the gels were washed 3 times in 2.5% (w/v) and treated with Triton X-100 for 30 minutes at room temperature to remove the SDS and then incubated in a reaction buffer containing 50 mM Tris (pH 7.4), 5 mM CaCl_2_ and 150 mM NaCl for 24 hours at 37°C, followed by staining with 2.5% (w/v) Coomassie blue in 30% (v/v) methanol and 10% (v/v) acetic acid. The MMP-2 and MMP-9 activities were detected as clear bands on a blue background with stained undigested gelatin.

### Immunocytochemical staining of cultured cells

Glass cover slips were sterilized by dipping them in 90% ethanol. After drying over a flame, each slip was placed into each well of 6-well culture plates. NSCLC cells (5 × 10^6^) in 2 mL of RPMI medium containing 10% FBS were seeded over the slip and grown at 37°C until 50-70% confluence reached. Following removal of the culture medium, cells in each well were rinsed twice with PBS and fixed with 3% paraformaldehyde (Sigma) in PBS and then incubated at room temperature for 20 minutes. After rinses with PBS for three times, cells were treated with a Triton X-100 buffer containing 50 mM Tris, 1 mM EDTA and 0.5% Triton X-100, pH 8.0 at room temperature for 15 minutes. Following washes with PBS, the slips were incubated with 10% normal horse serum to block nonspecific staining and then incubated with a mouse monoclonal antibody against COX Va (MitoScience, Eugene, OR) at a 1:100 dilution in a humid chamber at 4°C overnight. After washes, cells were incubated with a biotinylated horse anti-mouse IgG for 30 minutes and incubated with streptavidin-conjugated peroxidase for 30 minutes. Cells containing COX Va were visualized by incubation with 3,3-diaminobenzidine solution (0.3% hydrogen peroxide and 0.05% 3,3-diaminobenzidine) and counterstained with hematoxylin. Negative controls were prepared by substituting a normal mouse IgG as the primary antibody.

### Preparation of tissue core array

All of the pathologic sections were reviewed by one pathologist (T.Y.C.). Cores of tissue in demand were manually obtained from a tissue block by a 10-gauge syringe needle as described previously [18]. The cores were placed in a warm cast containing partially melted paraffin and arranged into a 6 x 8 array with one core at the upper-left corner removed as an orientation marker. Each array contained 47 cores with each patient sample from two to five cores, depending on the availability of tissue blocks. The positions of each core were recorded on reference sheets to facilitate data acquisition.

### Immunohistochemical staining of tumor tissues and evaluation of COX Va immunostaining

Immunostaining was performed by biotin-streptavidin-peroxidase procedures using a Vectastain ABC Kit (Vector Laboratories, Burlingame, CA). Fresh tumor tissues from patients with NSCLC and tissue core arrays were fixed in formalin and embedded in paraffin, to which sliced serial sections in 4 μm were mounted on glass slides, deparaffinized with xylene and dehydrated with alcohol according to the manufacturer’s instructions. Following retrieval of the antigen by heating the slice in a microwave for 2 minutes at 900W, the slides were incubated with 0.3% H_2_O_2_ solution in methanol for 30 minutes to block endogenous peroxidase. After rinses with PBS for three times, the tissues were treated with protease K (20 μg/mL a buffer containing 50 mM Tris, 1mM EDTA, 0.5% Triton X-100, pH 8.0) for 15 minutes at room temperature to expose the immunoreactive parts of COX Va on the mitochondrial membrane. Thereafter, the rest of the immunostaining procedures were conducted according to that described above.

Each stained core was evaluated according to the gross percentage of cells demonstrating nuclear and cytoplasmic immunoreactivity on the tumor part, and the assessment was accomplished independently by two examiners (W.L.C and K.T.K.). The scoring criteria for COX Va were as follows: 0, no appreciable staining in cells; 1+, appreciable nuclear staining with cytoplasmic staining in <25% of cells; 2+, appreciable nuclear staining with cytoplasmic staining between 25% and 50% of cells; 3+: appreciable nuclear staining with cytoplasmic staining in >50% of cells. Only 3+ staining was defined as COX Va overexpression or COX Va (+). Stage of disease progression was classified according to the 6th edition American Joint Committee on Cancer (AJCC) Staging System.

### Statistical analysis

The data were analyzed and are presented as mean ± SD (standard deviation). The difference between the compared groups was calculated by Mann-Whitney U test. Pathologic T, N, M status and tumor stage derived from new lung cancer staging system in the database were reviewed. The relationship between pathologic factors and COX Va expression status was analyzed by χ^2^ test (or two-tailed Fisher’s exact test when the expected number in any cell was smaller than 5 cases). A *P* value < 0.05 is considered statistically significant.

## Results

### Migration and invasion

**Figure 1:**
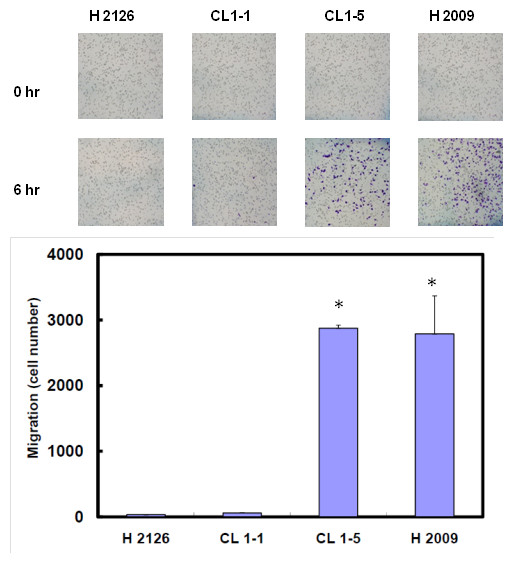
**Differences in migration ability among four NSCLC cell lines (H2126, CL1-1, CL1-5 and H2009).** Migration ability was assessed by Transwell assay. Each bar represents the mean ± SD (standard deviation) of the results from triplicates. The asterisk indicates a significant difference (*P* < 0.001) between either H2009 or CL1-5 and CL1-1 or H2009.

**Figure 2:**
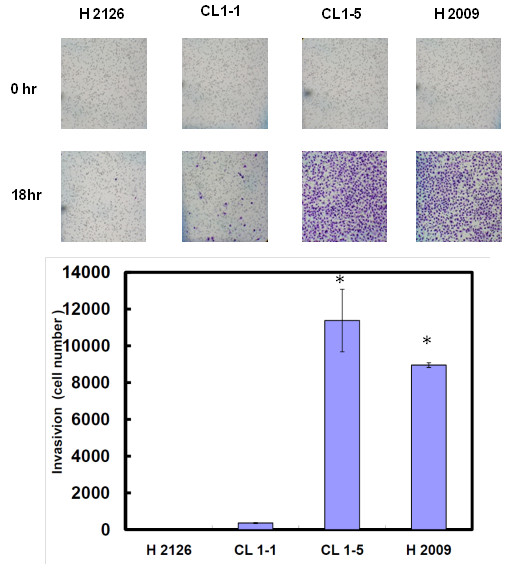
**Differences in invasion ability among four NSCLC cell lines (H2126, CL1-1, CL1-5 and H2009).** Invasion ability was assessed by Matrigel assay. Each bar represents the mean ± SD of the results from triplicates. The asterisk indicates a significant difference (*P* < 0.001) between either H2009 or CL1-5 and CL1-1 or H2009

We evaluated four NSCLC cell lines (CL1-1, CL1-5, H2126 and H2009) for their abilities in cell migration and invasion. As shown in Figures 1 and 2, those cell lines had stronger migration also had stronger invasion. Thus we divided them into low (H2126 and CL1-1) and high (CL1-5 and H2009) migration/invasion group.

### 2D Gel electrophoresis analysis

**Figure 3:**
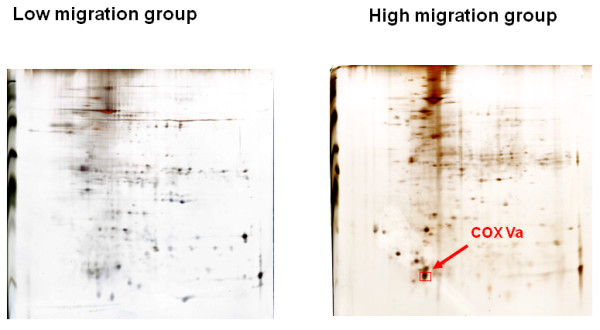
**A typical example of membrane-associated proteins analyzed by 2D-gel between low (CL1-1) and high (CL1-5) migration/invasion lung cancer cell lines.** A sample containing 200 μg of membrane proteins was initially run by isoelectrofocusing (pH 3 to 10 from left to right), which was followed by a conventional SDS-PAGE. Gels were then stained with a silver staining solution.

Next, we analyzed the membrane protein profiles between the high and low migration/invasion groups by 2D PAGE. A representative example of the comparison is depicted in Figure 3. The proteins overexpressed in high migration/invasion group were selected as targets since they might serve as potential biomarkers in a usual pathology laboratory. Following MALDI-TOF MS analyses, we identified 10 proteins, with their details listed in Table 1. Among them, one mitochondrial membrane-bound protein called cytochrome *c* oxidase subunit Va (COX Va), was of most interest because it was markedly overexpressed in tumorours tissues.

**Table 1:** Up-regulated proteins in highly migration/invasion NSCLC cell lines

Protein name	Molecular weight (kDa)	Location	Function
COX Va	16.7	mitochondria inner membrane	electron carrier activity
Caveolin-1	25	membrane	protein Tyrosine Phosphatase and G-protein–mediated signal transduction
Galectin-1	14	membrane	regulate apoptosis, cell proliferation and differentiation
Galectin-3	35	cytosol and Mitochondria inner membrane	cell differentiation
Glucose-regulated protein 78	78	endoplasmic reticulum	involved in the folding and assembly of proteins
Heat shock 70 kDa protein 8	70	endoplasmic reticulum	protein refolding and antiapoptosis,
Tyrosine-protein kinase 7; AXL	107	membrane	signal transducer
Trophinin	70	membrane	adhesion molecule
UDP-glucuronosyltransferase	56	endoplasmic reticulum	bilirubin conjugation
Vimentin	57	cytoskeleton	cellular component movement

### Characterization of molecular and protein expression of COX Va in NSCLC cell lines by RT-PCR, western blot and immunocytochemical staining

**Figure 4:**
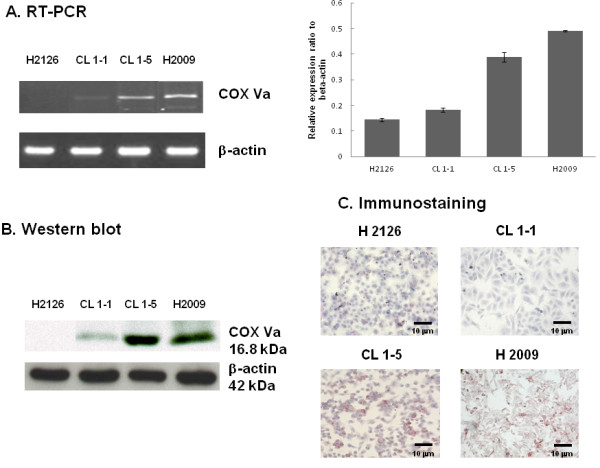
**Comparison of the expression of mRNA and protein of COX Va in NSCLC cell lines using RT-PCR, western blot and immunocytochemical staining.** (**A**) RT-PCR. A quantification plot is also illustrated. (n = 3). (**B**) Western blot. Each sample containing 10μg membrane proteins was loaded on a 15% SDS-PAGE. (**C**) Immunocytochemical staining was performed by using a monoclonal antibody against COX Va.

To evaluate whether this newly identified protein COX Va was overexpressed in situ in the cells with high migration/invasion, we conducted analysis for its mRNA expression using RT-PCR. Figure 4A shows that the cell lines in high migration/invasion group had greater COX Va mRNA levels as compared with those in low migration/invasion group. The higher expression in protein level was also documented by western blot, as shown in Figure 4B. To further confirm these observations, we determined the level of COX Va using an immunostaining in these four cell lines. Pre-immune serum or unrelated monoclonal antibody was used as a negative control. After treatment of the cells with Triton X-100, Figure 4C shows that there was a striking difference in immunostaining activity between the high and low migration/invasion cells. These results confirmed that the cell lines in high migration/invasion group had higher intensity of COX Va expression.

### Decreased migration/invasion of NSCLC cells by knocking down COX Va

**Figure 5:**
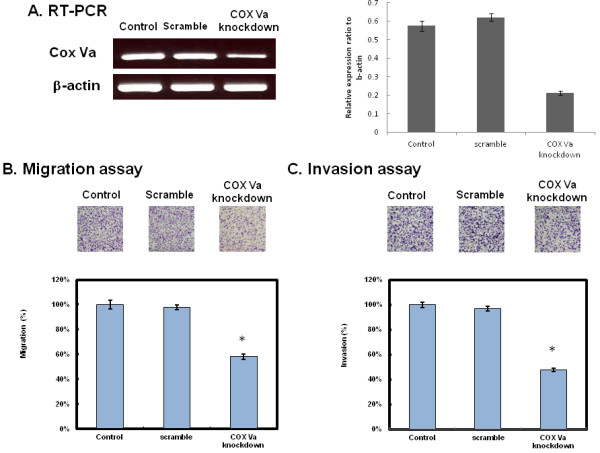
**The effect of COX Va gene knockdown on the migration/invasion of NSCLC cells.** (**A**) RT-PCR for COX Va in original H2009 cells (control), scramble and H2009 cells transfected with the shRNA against human COX Va. A quantification plot is also illustrated. (n = 3). (**B**) Migration activity of H2009 cells following transfection. Each bar represents the mean ± SD of the results from triplicates. (**C**) Invasion activity of H2009 cells following transfection. Each bar represents the mean ± SD of the results from triplicates. The asterisk indicates a significant difference (*P* < 0.01) between either original H2009 (control) or scramble and COX Va-knockdown cells.

To further testify whether COX Va plays a role in tumor cell migration/invasion, we constructed shRNA to knockdown COX Va at its mRNA level in the high migration/invasion group, with the H2009 cells as the representative. The result of RT-PCR (Figure 5A) shows that the expression of COX Va mRNA was substantially attenuated in COX Va-knockdown cells comparing with original H2009 (control) or scramble. Furthermore, cells with COX Va-knockdown had 42% decrease in migration and 52% decrease in invasion, respectively, as compared with original H2009 (control) or scramble (*P* < 0.01) (Figure 5B and 5C). These findings suggest that expression of COX Va was associated with migration/invasion of NSCLC cells.

### MMP assay

**Figure 6:**
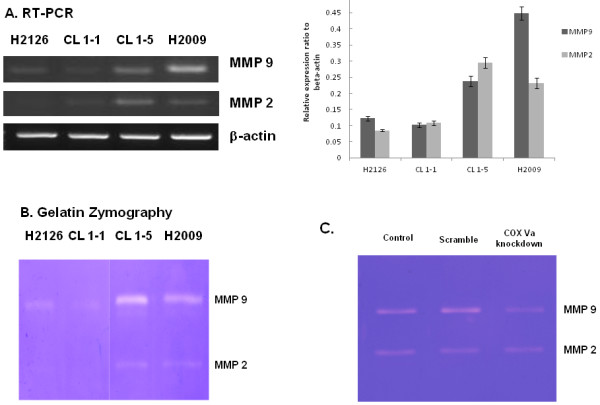
**Studies of MMP-2 and MMP-9 in NSCLC cells.** (**A**) RT-PCR for MMP-2 and MMP-9 in four NSCLC cell lines. A quantification plot is also illustrated. (n = 3). (**B**) Gelatin zymography of enzyme activity determined from concentrated culture media of four NSCLC cell lines after 48 hours of incubation. (**C**) MMP-2 and MMP-9 activities in original H2009 cells (control), scramble and COX Va-knockdown H2009 cells.

To look into the factors that were possibly linked to COX Va expression, we examined the expression of MMP-2 and MMP-9 in these four NSCLC cells. In addition to RT-PCR, we used gelatin zymography to assess the enzyme expression of MMP-2 and MMP-9. Figure 6A and 6B show that cell lines with high migration/invasion (CL1-5 and H2009) had stronger expression of MMP-2 and MMP-9 comparing to those with low migration/invasion (H2126 and CL1-1), in mRNA and enzyme activity level, respectively. These findings may partly account for why CL1-5 and H2009 had higher migration/invasion than did H2126 and CL1-1. Furthermore, the enzyme activity of MMP-2 and MMP-9 was also shown to be reduced in COX Va knockdowned H2009 cells (Figure 6C).

### Bcl-2 evaluation

**Figure 7:**
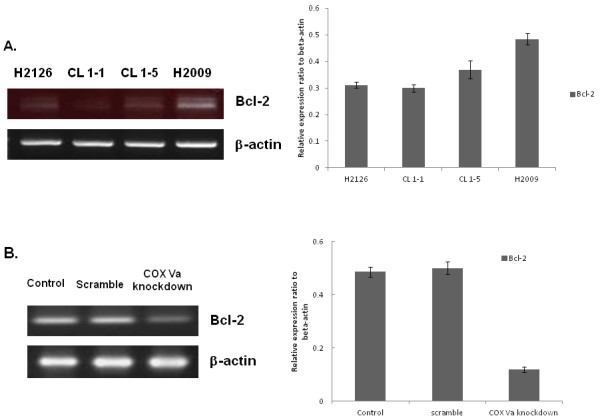
**Bcl-2 expression in NSCLC cells.** (**A**) RT-PCR for Bcl-2 in four NSCLC cell lines. A quantification plot is also illustrated. (n = 3). (**B**) RT-PCR for Bcl-2 in original H2009 cells (control), scramble and COX Va-knockdown H2009 cells. A quantification plot is also illustrated. (n = 3).

Bcl-2 and its family proteins are integral membrane proteins located mainly on the outer membrane of mitochondria and have been reported to interact with cytochrome *c*[19,20]. Therefore, the mRNA expression of Bcl-2 in four cell lines was also evaluated. As shown in Figure 7A, the cell lines in high migration/invasion group (CL1-5 and H2009) had higher expression of Bcl-2 mRNA. After knocking down COX Va expression, the H2009 cells with COX Va-knockdown also had lower expression of Bcl-2 mRNA (Figure 7B).

### Expression of COX Va in surgically resected tissues of patients with NSCLC

**Figure 8:**
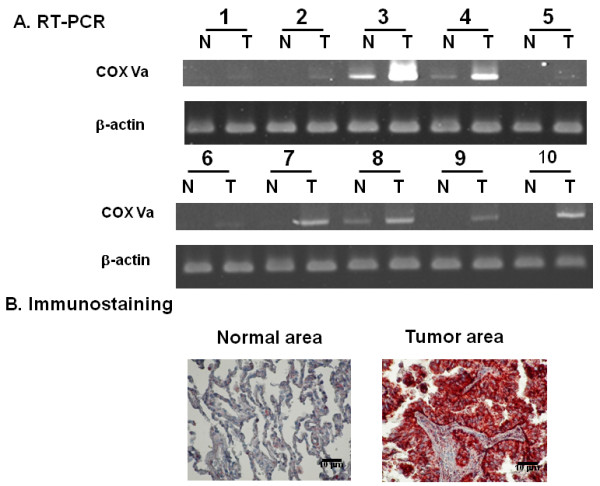
**Molecular expression and immunostaining of COX Va in pairs of surgically resected lung tissues from 10 patients with NSCLC.** (**A**) RT-PCR. (**B**) Immunohistochemical staining. A typical example depicts a positive immunostaining in tumorous tissues (right), but not in non-tumorous tissues (left).

Paired tumorous and non-tumorous (stromal area) fresh frozen specimens from 10 subjects with NSCLC were randomly selected to evaluate the mRNA expression of COX Va by RT-PCR. It revealed that the expression condition of COX Va mRNA was variable, with greater level in tumorous area in most cases (Figure 8A). With respect to immunostaining in the fresh resected tissues, it was of interest to note that COX Va can only be stained by treating the tumorous tissue with Triton X-100 plus protease K. A typical example showing tumorous area being highly positive is depicted in Figure 8B. To our surprise, we found that non-tumorous areas were essentially negative for COX Va, which is similar to negative controls stained with normal mouse serum (data not shown). However, it should be reminded that areas without reactivity in immunostaining do not necessarily indicate a lack of biosynthesis of COX Va. For example, expression of COX Va mRNA in non-tumorous tissue could still be observed in subject No.3, 4 and 8 (Figure 8A).

Using tissue core array, we also studied the immunoreactivities of COX Va in formalin-fixed, paraffin-embedded specimens and analyzed their pathologic significance in 250 consecutive patients with adenocarcinoma. Strong COX Va expression was found in 54.8% (137/250) of patients and correlated positively with the N status (*P* = 0.032), but without significant correlation with T status (*P* = 0.667), M status (*P* = 0.802) or stage (*P* = 0.232) (Table 2). During follow-up, strong COX Va expression was associated with distant metastasis significantly (*P* = 0.033) but not local recurrence (*P* = 0.465). However, COX Va expression was not associated with either overall survival (*P* = 0.329) or disease-free survival (*P* = 0.189). The median survival was 65.1 months for strong COX Va patients, and 69.8 months for weak COX Va patients.

**Table 2:** Relationship between COX Va expression and clinicopathologic parameters

Parameters	COX Va (+), n = 137	COX Va (-), n = 113	*p* value
T status			0.667
T1	27	31	
T2	98	68	
T3	1	5	
T4	11	9	
N status			0.032*
N (-)	83	83	
N (+)^a^	54	30	
M status			0.802
M (-)	130	108	
M (+)	7	5	
Stage			0.232
I	76	72	
II	15	9	
III	39	27	
IV	7	5	
Local yes	9	5	0.465
recurrence no	128	108	
Distant yes	25	10	0.033*
metastasis no	112	103	

^a^N(+) includes N1and N2 but without N3.

## Discussion

Metastasis remains the major problem in managing NSCLC, and invasion is the first step of metastasis. Thus understanding more about invasion will provide us greater opportunities to treat NSCLC. Previous reports [21,22] have concluded some markers regarding metastasis and invasion in lung cancer, such as MMPs, vascular endothelial growth factor (VEGF) and cyclooxygenase-2 (COX-2). Among these markers, some therapeutic agents derived from them had been proved to be useful in clinical practice [23-25].

In this study, using NSCLC cell lines with different abilities of migration/invasion, we distinguished a membrane-bound protein that was associated with migration/invasion of NSCLC by proteomic approach. Totally 10 membrane-associated proteins were identified after 2D gel electrophoresis and subsequent MALDI-TOF MS studies, and COX Va was of particular interest because of its exclusive presence in the surgical specimens from NSCLC patients. Further experiments supported the assumption that COX Va was associated with migration/invasion of NSCLC.

First, knockdown of COX Va gene expression suppressed migration/invasion of NSCLC cells effectively (Figure 5). Second, the cells expressed higher level of COX Va also had higher expressions and activities of MMP-2 and MMP-9 (Figure 6A and 6B), which had been reported to be related to migration and invasion in different types of tumor cells [16,17,26-28]. Meanwhile, such a correlation was confirmed again in COX Va knockdown study (Figure 6C). Third, in 250 surgically resected adenocarcinomas, overexpression of COX Va correlated positively with the N status (lymph node metastasis) and distant metastasis. These data highlighted the clinicopathologic role of COX Va.

Structurally, COX is a mitochondrial transmembrane enzyme consisting of 13 subunits in mammals, among which only the three largest subunits (I-III) are encoded by mitochondrial DNA. COX is a rate-limiting enzyme present as a dimeric unit participating at the terminal step in respiratory chain to reduce molecular oxygen into water and pump protons across the inner mitochondrial membrane [29-32]. A recent study showed that high levels of COX activity and mitochondrial respiration in tumor cells lead to an overexpression of Bcl-2 [33]. Furthermore, it has been reported that Bcl-2 directly binds to COX to promote survival of cancer cells by maintaining a slight pro-oxidant state through elevated mitochondrial respiration during basal conditions [34]. In the same study, a protein-protein interaction between COX Va and Bcl-2 was also documented by coimmunoprecipitation.

Although the causal relationship between COX Va and MMP-2/MMP-9 could not be demonstrated in the current study, some speculation may be provided. From our current data, the cell lines in high migration/invasion group (CL1-5 and H2009) also had higher expression of Bcl-2 and knocking down COX Va was associated with decreased expression of Bcl-2. It implied that the activity of COX Va was parallel to the expression of Bcl-2. Previously, it has been reported that Bcl-2 promotes invasion and lung metastasis by inducing MMP-2 in NSCLC cells [35] and coexpression of Bcl-2 and N-Myc induces MMP-2 secretion and activity in human neuroblastoma cells [36]. In an animal study, down-regulation of Bcl-2 was observed to be associated with down-regulation of both MMP-2 and MMP-9 expression [37]. Thus we consider that overexpression of COX Va may result in increased expression of MMP-2 and MMP-9 via collaboration with Bcl-2. Nevertheless, further investigations are needed to elucidate the mechanistic insight.

## Conclusions

To sum up, we identified a membrane-bound protein COX Va from NSCLC cells with different abilities of migration/invasion and confirmed its relationship to migration/invasion by several *in vitro* experiments. The clinicopathologic role of COX Va was also demonstrated by its positive correlation with lymph node metastasis in surgically resected adenocarcinomas plus prediction of distant metastasis during follow-up. Meanwhile, expression of COX Va was found parallel to expression of Bcl-2, MMP-2 and MMP-9. All these data suggested that COX Va plays a role in migration/invasion of NSCLC cells and is worth of further study to serve as a therapeutic target in NSCLC.

## Competing interests

The authors declare that they have no competing interests.

## Authors’ contributions

WLC initiated the study, performed proteomic part of the experiments and drafted the manuscript. KTK performed the immunohistochemistry part of the experiments and assisted in the writing of the manuscript. CLC performed migration and invasion parts of the experiments. CHW, TYC and YHW contributed to experimental design and data discussion. LSW designed and supervised the experiments, and proofed the manuscript. All authors read and approved the final draft of the manuscript.

## Pre-publication history

The pre-publication history for this paper can be accessed here:

http://www.biomedcentral.com/1471-2407/12/273/prepub
